# *Paepalanthus rectifolius*, a new name in Eriocaulaceae (Poales)

**DOI:** 10.3897/phytokeys.10.2591

**Published:** 2012-03-09

**Authors:** Livia Echternacht, Marcelo Trovó, Paulo Takeo Sano

**Affiliations:** 1 Laboratório de Sistemática Vegetal, Departamento de Botânica, Instituto de Biociências, Universidade de São Paulo, Rua do Matão 277, CEP 05508-900, São Paulo, SP, Brasil; 2 UMR 7207 CNRS MNHN UPMC, Centre de Recherche en Paléobiodiversité et Paléoenvironnements, MNHN, 57 rue Cuvier, CC48, F-75005, Paris, France; 3 Departamento de Botânica, Instituto de Biologia, Universidade Federal do Rio de Janeiro, CCS, Bloco A1, Cidade Universitária, Ilha do Fundão, CEP 21941-590, Rio de Janeiro, RJ, Brasil; 4 Evolution and Biodiversity of Plants, Faculty for Biology and Biotechnology. Ruhr University Bochum, D-44780 Bochum, Germany

**Keywords:** Brazil, Goiás, Nomenclature, *Syngonanthus*, Taxonomy

## Abstract

*Syngonanthus weddellii* var. *gracilis* Moldenke, (1973) was described very briefly based on a single collection. A careful analysis reveals that this variety has dimerous flowers, free petals of the pistillate flower and bifid stigmatic branches. It is therefore misplaced in *Syngonanthus* Ruhland (1900). We transfer it to *Paepalanthus* Mart. (1834) at the species level, as it is distinct from morphologically similar species: *Paepalanthus flaccidus* (Bong.) Koern. (1863), *Paepalanthus trichophyllus* (Bong.) Koern. (1863), and *Paepalanthus strictus* Koern. (1863). The epithet gracilis is no longer available, hence, we have coined the name *Paepalanthus rectifolius*. We also provide a full description, illustrations, a distribution map, and pertinent comments.

## Introduction

*Paepalanthus* Mart. (1834) is one of the largest genera of Eriocaulaceae, comprising ca. 400 species (Giulietti and Hensold 1990, Stützel 1998). It is differentiated from *Syngonanthus* Ruhland (1900), which encompasses ca. 130 species, primarily by its completely free petals of the pistillate flower (Ruhland 1900, 1903). Both genera are widely distributed throughout the Neotropics, with a few species occurring in Africa, Central America, and in the case of *Syngonanthus*, also in North America (Koernicke 1863, Ruhland 1903, Giulietti and Hensold 1990, Stützel 1998).

In the last century, Harold Norman Moldenke published hundreds of new Eriocaulaceae taxa, most of them based on the specimens of his personal herbarium, now hosted in the LL herbarium, and on the specimens deposited in the NY herbarium. Many of these descriptions are quite short, especially those from taxa below the species level. Thus, the identification of such taxa is problematic, relying obligatorily on the analysis of the type specimens. Recent analysis of these specimens leads to the conclusion that *Syngonanthus weddellii* var. *gracilis* Moldenke, (1973) is misplaced within *Syngonanthus* and should be considered a distinct species in *Paepalanthus*. In order to solve this issue, we propose the following nomenclatural changes. We also provide a full description, comments, illustrations, and a distribution map.

## Taxonomy

### Paepalanthus rectifolius

Trovó, Echtern. & Sano nom. nov.

urn:lsid:ipni.org:names:77118000-1

http://species-id.net/wiki/Paepalanthus_rectifolius

Fig. 1

#### Replaced name:

*Syngonanthus weddellii* Moldenke var. *gracilis* Moldenke, Phytologia 25: 224. 1973. TYPE: BRAZIL. Goiás. Pirenópolis: Serra dos Pirineus, ca. 18 km E of Pirenópolis town, 1000 m alt., 15 Jan. 1972, H. S. Irwin, W. R. Anderson, M. Stieber & E. Y. Lee 34259 (holotype, LL!; isotype, NY!).

Herbs, 20–40 cm long. Aerial stem 10–20 cm long, pilose with long curled filamentous trichomes ca. 1 cm long, bearing distal inflorescences; after the flowering period, the stem elongates and ramifies distally to the inflorescences, giving rise to other inflorescences in the next fertile period. Leaves spirally disposed along the elongated stem, persistent, linear, flat, patent, 2.0–4.0 × 0.1–0.2 cm, villous in both surfaces, with pedicellate filamentous erect to curled trichomes ca. 0.7 cm long, sheath enlarged, up to 2–3 mm, semi-amplexicaul, apex acute to acuminate. Spathes 2.5–3.5 cm long, abaxial surface pubescent as the leaves, oblique opening, apex acute. Scapes 10–45 per fertile branch, 15–25 cm long, pubescent with short adpressed simple trichomes, early glabrescent. Capitula 5–8 mm diam., spherical; involucral bracts in 5–8 series, oblong, concave, ca. 3.0 × 1.0 mm, external series completely glabrous in both surfaces, internal series densely tufted and ciliated at the apex, with trichomes ca. 0.3 mm long, with clavate apical cells, golden, apex obtuse; receptacle semi-spherical, hairy. Flowers dimerous, ca. 150 per capitulum, ca. 4 times more staminates than pistillates. Floral bracts linear, flat, ca. 1.5 mm long, hairy toward the apex to glabrescent, trichomes with clavate apical cells, brown to dark, apex acute. Staminate flowers ca. 1.5–2.0 mm long, including the pedicel; pedicel ca. 0.5 mm long, with long (ca. 1 mm) trichomes; sepals navicular, ca. 1.0–1.5 mm long, hairy toward the apex, soon glabrescent, trichomes with clavate apical cells, brown to dark in the apex, cream at the base, apex truncate, membranaceous; antophore ca. 0.5 mm long, cream, membranaceous; corolla tubular, ca. 1.0 mm long, glabrous, with the same color as the sepals, membranaceous, involute after anthesis; stamens ca. 1.0 mm long, anthers white; pistillodes 2, ca. 0.1 mm long, papillose, hyaline. Pistillate flowers ca. 1.5–2.0 mm long, including the pedicel; pedicel ca. 0.5 mm long, with trichomes ca. 1 mm long; sepals navicular, ca. 1.0–1.5 mm long, hairy toward the apex, soon glabrescent, trichomes with clavate apical cells, brown to dark in the apex, cream at the base, apex truncate, membranaceous; petals free, ca. 1.0 mm long, densely pilose toward the apex, trichomes with clavate apical cells, hyaline, membranaceous; gynoecium ca. 0.5 mm long, ovary ca. 0.2 mm long, style ca. 0.2 mm, appendages ca. 0.05 mm long, hyaline, inserted at the same point of the stigmatic branches, stigmatic branches 0.2 mm long, bifid. Seeds not seen.

**Figure 1.**
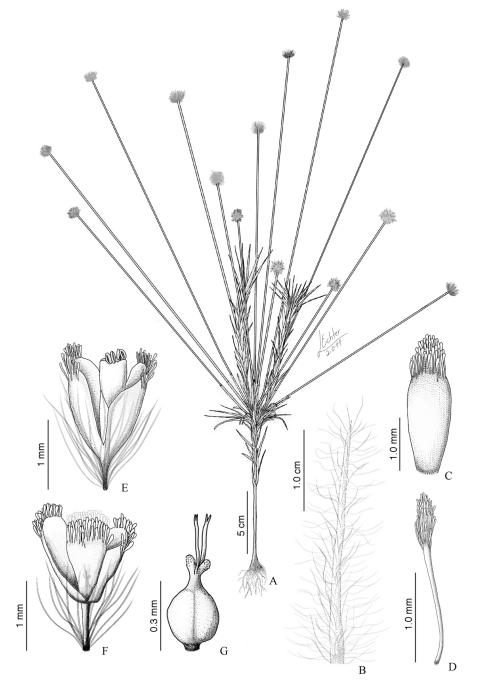
*Paepalanthus rectifolius* Trovó, Echter & Sano **A** Habit **B** Leaf detail **C** Involucral bract from the internal series, abaxial surface **D** Floral bract abaxial surface **E** Staminate flower **F** Pistillate flower **G** Gynoecium. Drawn from the type collection (*H. S. Irwin*, *W. R. Anderson*, *M. Stieber* & *E. Y. Lee 34259*, LL).

#### Comments.

**Figure 2.**
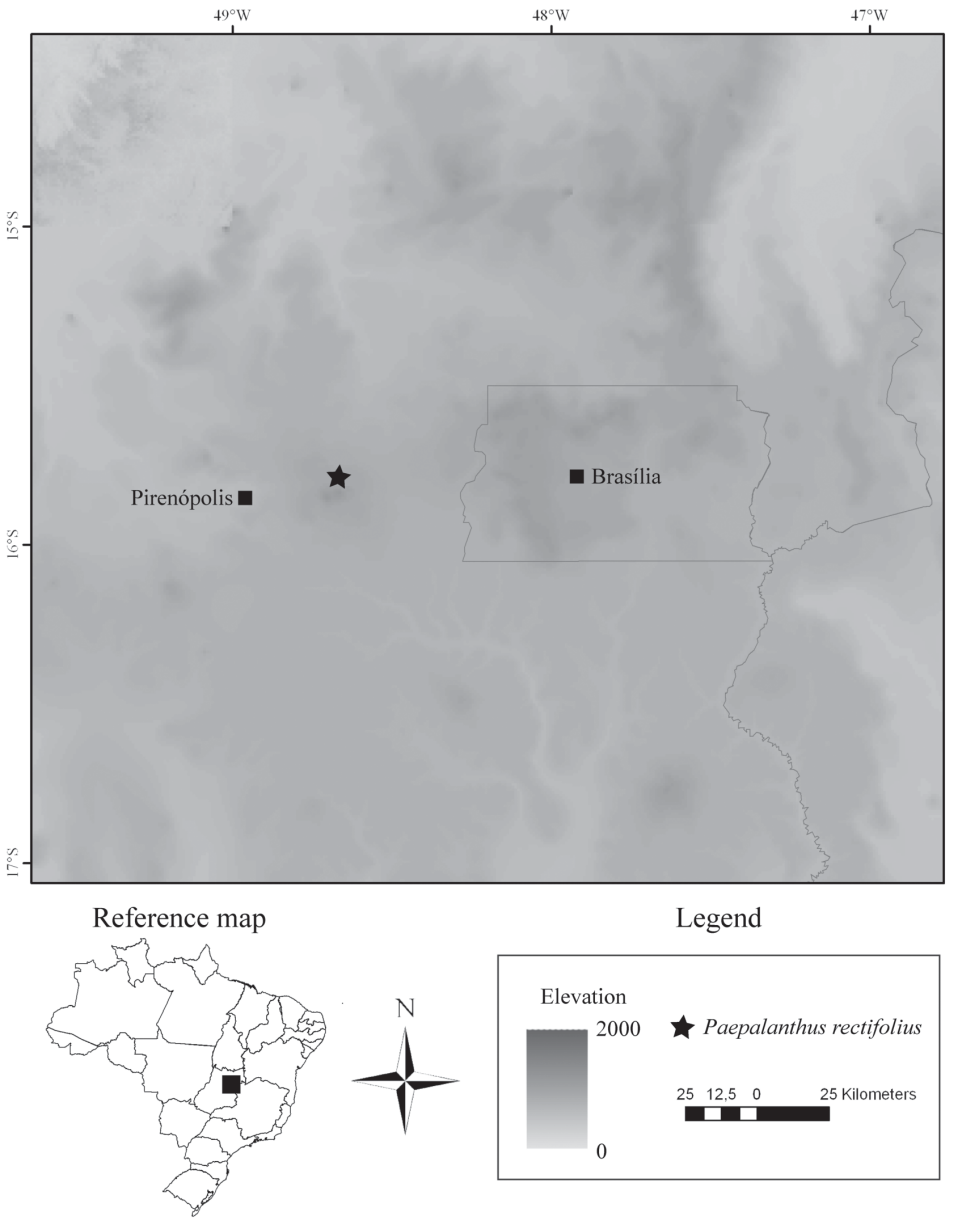
*Paepalanthus rectifolius* Trovó, Echter & Sano distribution map.

*Syngonanthus weddellii* var. *gracilis* Moldenke was misplaced in *Syngonanthus* as this taxon has truly free petals on the pistillate flower and bifid stigmatic branches. It is therefore transferred at the species level to *Paepalanthus*, a genus encompassing such morphological traits. The epithet *gracilis* ,however, has been previously used in *Paepalanthus* by Koernicke (1863). Thus, since it is not available, we propose the new name *Paepalanthus rectifolius*, referring to straight and ascending leaves, which differ the species from the most similar ones. *Paepalanthus rectifolius* is known only from the type specimens collected in the mountains east of Pirenópolis, in the Serra dos Pirineus, Goiás, Brazil (Fig. 2). During our research on Eriocaulaceae systematics, we studied collections from several herbaria (B, BHCB, BM, BR, BRLU, C, CESJ, ESA, ESALQ, F, G, HUEFS, INPA, K, L, LE, LL, M, MO, NY, OUPR, OXF, P, R, RB, S, SP, SPF, UEC and UPS; acronyms in Thiers continuously updated). However, despite the presence of several collections from the Serra dos Pirineus, we did not find other specimen of *Paepalanthus rectifolius* besides the type. Individuals with immature as well as old inflorescences were collected in January.

The morphologically related species are *Paepalanthus flaccidus* (Bong.) Koern. (1863), *Paepalanthus trichophyllus* (Bong.) Koern. (1863), and *Paepalanthus strictus* Koern. (1863), three species with dimerous flowers recently excluded from *Paepalanthus* sect. *Diphyomene* Ruhland (1903) (Trovó and Sano 2010). As mentioned above, *Paepalanthus rectifolius* differs from these three species by its ascending and straight leaves (vs. recurved). It is easily differentiated from *Paepalanthus strictus* and *Paepalanthus trichophyllus* by its linear leaves (vs. lanceolate), golden involucral bracts (vs. dark castaneous), and linear floral bracts (vs. oblong). *Paepalanthus flaccidus* is the most similar species, due to the linear leaves, to the golden involucral bracts, whose internal series might also be tufted in the apex, and to the villous pubescence, with long pedicellate trichomes. Both may be considered sympatric as they are reported to Goiás and occur in the same habitats. *Paepalanthus rectifolius* is distinguished from *Paepalanthus flaccidus* by its much longer leaves (2.0–4.0 cm vs. 0.5–1.5 cm), linear floral bracts (vs. oblong), and staminate flowers without lobes (vs. markedly lobed).

## Supplementary Material

XML Treatment for Paepalanthus rectifolius
